# Intergenerational and intragenerational preferences in a developing country to avoid climate change

**DOI:** 10.3389/fpsyg.2023.1098382

**Published:** 2023-02-03

**Authors:** Shahzad Alvi, Verda Salman, Fazal Un Nisa Bibi, Naima Sarwar

**Affiliations:** School of Social Sciences and Humanities, National University of Sciences and Technology (NUST), Islamabad, Pakistan

**Keywords:** inter-generation, intra-generation, climate change, motivation, preferences, sustainability

## Abstract

Intergenerational and intragenerational approaches to climate change take into account the actions taken by the current generation to maintain or improve the climate, which is advantageous to both the present and future generations. Climate-friendly initiatives primarily benefit future generations, with current generations receiving lesser benefits. Self-interest can hinder the management of shared resources, as seen in the “tragedy of the commons” concept, where individuals benefit from defecting, but society bears the consequences of it. This study used three different time horizons to determine the inter- and intra-generational preferences of groups of human subjects for preventing hazardous climate change. We looked at how groups of participants responded in scenarios that varied in motivation, income, social pressure, and learning opportunities. For this purpose, we conducted two group experiments framed around climate change where participants could choose to cooperate for a noble cause: tree plantations. Its rewards are delayed by several years and probably a few decades (intergenerational discounting), where future generations will be the big beneficiaries. There were two more options: the first one delayed the reward by 1 week, and the second was delayed by seven weeks (intragenerational discounting). We found that intergenerational discounting was high when the groups had free will and motivation. Further, it is revealed that having more money does not play a significant positive role in long-term climate sustainability in a developing country; however, it does, but not as much as motivation and free will do.

## 1. Introduction

Humans have much to worry about regarding climate change. Scientists, researchers, and policymakers agree that “dangerous climate change” is defined as a global mean temperature rise of more than 2°C above pre-industrial levels. This implies that greenhouse gas emissions must be halved by 2050 relative to their current levels (Meinshausen et al., 2009). Investing in lowering carbon emissions currently indicates shifting our consumption away from ourselves—whoever “we” are the ones who are making these sacrifices—for the benefit of individuals in the future (Schelling, 1995). In terms of intergenerational justice, or the idea that people living now have responsibilities to people living in the future, climate change brings up some of the most important questions, such as which risks people living now are allowed to put on people living in the future and how natural resources can be shared fairly (Khattak et al., 2022; Nawaz et al., 2022).

People with a future-orientation are often more involved in environmental preservation than people with a present-orientation, suggesting a relationship between time orientation and pro-environmental attitudes and behaviors (Alvi and Khayyam, 2020; Alvi et al., 2020). Temporal discounting tasks, which require participants to select between smaller, more immediate rewards and bigger, more delayed benefits, are often used to test time preferences (Green et al., 1994; Beckerman and Hepburn, 2007; Frye et al., 2016).

Self-interest undercuts the management of shared resources in “the tragedy of the commons,” a phrase frequently used to refer to global environmental challenges; the advantages of defection are individual, while the consequences of defection are shared (Hardin, 1968). A public goods game that allows players to contribute all or part of their operational funds to a common pool can be used to imitate the tragedy of the commons. Regardless of whether and how much they invested, all members receive an equal share of the returns once the total investments are multiplied by a multiplier, often “2”. Defecting is profitable, but if everyone justifies it in this manner, the group forfeits the invaluable public good. On the contrary, if everyone had contributed, everyone would have benefited. The hallmark of social problems is this conflict of interest between the individual and the collective (Dawes, 1980). Insights into human behavior can be gained from public-goods experiments which demonstrate that adding punishment, reputation, or their interaction, as well as the threat of shame or the promise of honor and social pressure, can increase cooperation (Milinski et al., 2002; Rockenbach and Milinski, 2006; Gachter et al., 2008; Jacquet et al., 2011; Khan et al., 2021). On the other hand, individuals can learn that free riding is a dominant behavior in finitely repeated public goods games and decide to contribute less in the later rounds of the game (Andreoni, 1988). Nevertheless, games with a dominant strategy equilibrium converge to Nash equilibrium under alternative treatments (Grimm and Mengel, 2012).

A recent study by Milinski et al. (2008) shows that despite significant loss probability, humans frequently fail to manage group risk. To avoid having a 90% probability of losing their remaining assets, only 5 out of 10 groups were able to attain a target amount, which required 50% of the available cash (Milinski et al., 2008). Even if the experimental design of collective-risk studies has been positive thus far, these failures nonetheless happened because participants immediately after the trials experienced both the advantages of defection and the incentives of collaboration (Tavoni et al., 2011). Gains from defecting can frequently be achieved rapidly in the real-world collective-risk climate change problem, whereas the benefits of cooperating may take decades to materialize (Schelling, 1995). Therefore, a preference for advantages that come closer to the present, often known as “temporal discounting,” is another social characteristic that complicates collective-risk decision-making.

According to the discounting theory, receiving a smaller payment right away is frequently more valuable than receiving a larger benefit later. Because there is a chance that the benefit will not be realized or that the beneficiary will not survive to enjoy it, rewards in the present are worth more than rewards in the future. Although competition among individuals is one reason why resources are not managed sustainably (Hardin, 1968), overexploitation of resources is likely even under private control due to high rates of discounting (Clark, 1973), and this likelihood increases when future generations benefit from cooperation (Sumaila and Walters, 2005). All of the animal kingdoms exhibit high discount rates (Madden and Bickel, 2010). Depending on whether a reward is immediate or delayed, various areas in the human brain are activated, suggesting that imagining the future involves a separate cognitive function (McClure, 2004).

Motivation is a mechanism that may positively impact a person's decision-making power by directing him/her toward the target (Reckless et al., 2013). Marketers use different types of self-serving and self-sacrificing strategies to get support for charities (Bock et al., 2018). Campaigns designed to create awareness about a particular cause create feelings of empathy among the public and consequently increase charitable giving (Basil et al., 2006). Breeze and Dean (2012) found that showing a “disadvantaged group” as people who are helpless against natural forces makes people more likely to give money to charity.

People contribute to campaigns because it is a pleasurable experience that confirms or creates a positive self-image of helpfulness, being a good citizen, or being influential (Van Leeuwen and Wiepking, 2013). Furthermore, in addition to the “warm glow” effect, individuals may also seek to be perceived by others as benevolent and empathetic toward those in need. As a result, individuals may be inclined to donate to charitable organizations and causes more frequently due to social pressures (Bursztyn and Jensen, 2017).

We used three alternative time horizons to determine the inter- and intra-generational preferences of groups of human subjects for preventing hazardous climate change. We looked at how groups of participants responded in scenarios that varied in motivation, income, social pressure, and learning opportunities. In contrast to past studies, which largely focused on individual and group temporal discounting (Frederick et al., 2002; Jacquet et al., 2013), we looked at how discounting works in group settings when motivation is present. This study contributes in a novel way by investigating the function of social pressure, the learning effect, and motivation in collective-risk environmental games. In addition, we investigated the contribution patterns when agents must use an endowment as opposed to their own (earned) income.

## 2. Research method

The term “sustainable development” refers to a method of meeting present-day requirements without jeopardizing the ability of future generations to do the same. However, intergenerational fairness in the philosophy of justice begins with the basic concern for intergenerational distribution, namely that it would benefit current generations (Rawls, 2004). The deterioration of the climate not only exacerbates existing poverty but also limits the opportunities of future generations, resulting in intergenerational injustice. This effect is more pronounced over long time horizons, and it becomes crucial in decisions related to spending on climate change mitigation, which may be uncertain in terms of both magnitude and timing (Tietenberg and Lewis, 2018). Individuals are more likely to contribute to climate preservation if they have a future-oriented mindset (Alvi et al., 2020), are motivated (Basil et al., 2006), have exposure to social pressure (Khan et al., 2021), and have a windfall endowment (Cherry et al., 2005). The contributions are also affected if the game is repeated a finite number of times as players learn from the strategies and actions of others, are known as the learning effect Ostrom (2000), and understand intragenerational and intergenerational discounting (Sumaila and Walters, 2005).

In the present study, we analyzed intragenerational and intergenerational preferences through an experiment. A total of 44 participants were included in the study, with 22 participants in each experiment. All participants are students at the National University of Sciences and Technology. The consent of each participant was obtained before the experiment.

### 2.1. Experimental design

*Experiment 1*: A group consisting of two participants, A and B, was each given an initial amount of (Pak rupees) Rs. 4,000. The participants were required to invest in a “climate account” where contributions could be Rs. 0, Rs. 200, or Rs. 400; the decision of each participant was visible to the members of his or her group but not revealed to other groups. The participants were informed before the collection that their contributions would be used for climate protection. Decisions were visible to the group members at each round because this builds trust and increases the chances of achieving the target (from a behavioral point of view). Moreover, there are defectors in the game, too; thus, decisions were revealed to compensate for less cooperative group members (Jacquet et al., 2013). Social pressure is also a major factor in revealing decisions to group members, as adding punishment, reputation, or their interaction, and the threat of shame or the promise of honor, can increase cooperation (Milinski et al., 2002; Rockenbach and Milinski, 2006; Gachter et al., 2008; Jacquet et al., 2011; Khan et al., 2021). The same procedure was repeated in the subsequent 10 rounds. The purpose of extending this game to several rounds was to check whether the outcome converges to the Nash equilibrium of defection or not; players may decide to reduce their contribution if they observe defection as a dominant strategy (Andreoni, 1988; Grimm and Mengel, 2012).

Participants were informed of the investment reward after completing 10 rounds and that each group's investment was multiplied by a factor of two. The participants were then given three options for receiving their actual investment and rewards. First, they can get the amount after a local media campaign for climate protection for 1 week so that that the period could allow the use of the participant's investment amount (T1). Second, they can get the amount after seven weeks, which would be used for national-level campaigns (T2). Third, the participants would not receive their amount, which would be used for tree plantations (T3). The responses were noted, and participants were paid according to their preferred option. Individuals work to maximize their own benefits, eventually depleting the natural resource, as in the tragedy of the commons, but this time it is examined how they would behave in a group setting. The purpose of giving reward information after completing 10 rounds is to check the group's behavior and determine whether they will go for intragenerational or intergenerational discounting.

*Experiment 2*: This experiment differs from the first one in three aspects. (i) The participants were not given any amount to invest. In this experiment, they were asked to give some amount according to their free will in 10 rounds, and the upper threshold amount was Rs. 8,000 in the sum of all rounds. The goal of not providing any funds to the experiment participants was to see if the source of endowment (windfall or own/earned) affected the contribution to the climate account. (ii) The total investment of each group in the climate account was not doubled. (iii) All the participants were provided with information about the significance of climate change and its crucial effects on our daily lives and future generations. The participants were motivated by information about how fast the world is moving toward drastic climate change and, more importantly, how the personal preferences of individuals can make a big difference. It is important to note that, while transient emotions affect decision-making (Andrade and Ariely, 2009; Qiao-Tasserit et al., 2017), the impact of transient information on choices is not profound (Ahituv et al., 1998). On the other hand, transient scenarios like creating awareness about climatic uncertainties can influence climate adaptation decisions (Haasnoot et al., 2015).

Individuals were later informed that their funds would be used for media advertising or tree planting. Then, we informed them about returning the amount, which was to be given in three options after the contribution was made. First, the original amount would be given after 1 week (T1). In the second option, the original amount would return after seven weeks (T2). Third, all the amounts were to be used in the tree plantation. Participants were free to choose from all three options. T1 and T2 reflect participants' concerns for the current generation, while T3 is associated with more noble concerns for future generations. It can be argued that investing in climate campaigns impacts the actions and choices of people regarding climate preservation, which is also beneficial for future generations. This study, however, takes a limited account of the awareness campaigns restricted to the present generation only; this might be considered a limitation of the study.

## 3. Results and discussion

**Figure 1:**
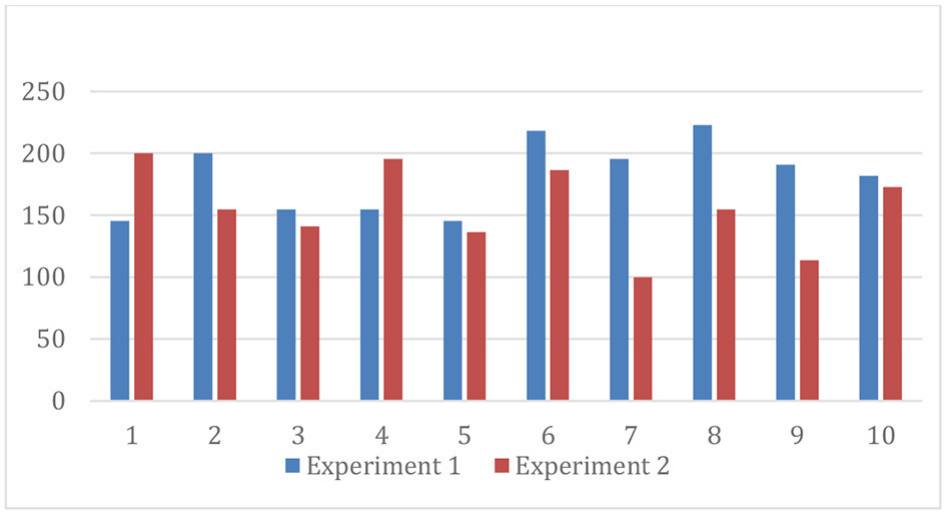
Average contributions in each round.

**Figure 2:**
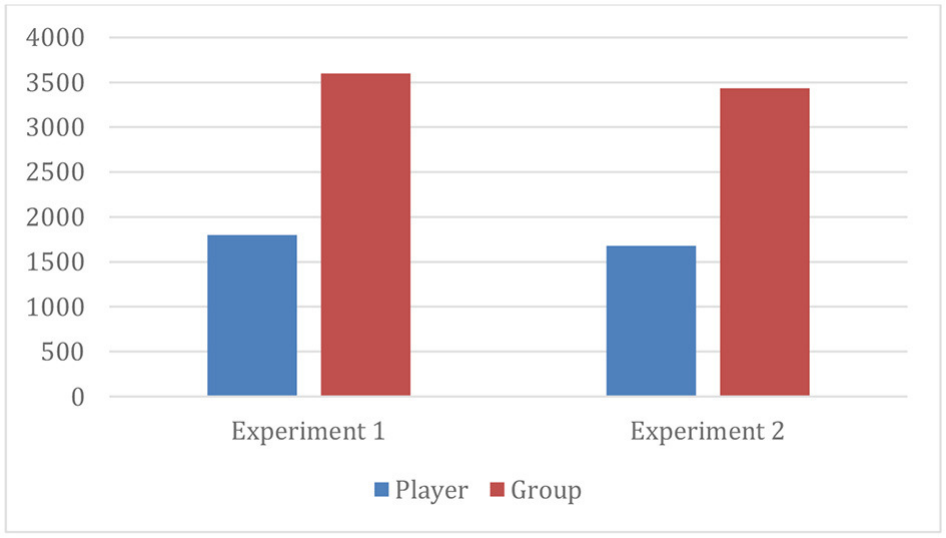
Average total contributions.

Tables 1, 2 present the contributions by each player in each round of experiments, while Figure 1 reveals the mean contributions in each round of experiments 1 and 2. It is evident that the mean contributions in each round of the two experiments follow a mixed trend; players are not visibly following any fixed strategy. In certain rounds, contributions are increased; social pressure is the reason. The decline in giving in the later rounds can be attributed to the learning effect; players know that noncooperation is the dominant strategy (Andreoni, 1988; Khan et al., 2021). Figure 2 shows the average total contribution by players and groups in each experiment. Average contributions in experiment 1 are higher as compared to experiment 2. This finding is supported by the literature, which suggests that people are more generous and risk-takers when they receive a windfall endowment rather than an earned one (Carlsson et al., 2013; Dankov and Servátka, 2015; Li et al., 2019).

**Table 1:** Results of 10 rounds in experiment I.

**Group 1**		**Group 2**		**Group 3**		**Group 4**		**Group 5**		**Group 6**		**Group 7**		**Group 8**		**Group 9**		**Group 10**		**Group 11**	
**(Pak Rupee)**		**(Pak Rupee)**		**(Pak Rupee)**		**(Pak Rupee)**		**(Pak Rupee)**		**(Pak Rupee)**		**(Pak Rupee)**		**(Pak Rupee)**		**(Pak Rupee)**		**(Pak Rupee)**		**(Pak Rupee)**	
**A**	**B**	**A**	**B**	**A**	**B**	**A**	**B**	**A**	**B**	**A**	**B**	**A**	**B**	**A**	**B**	**A**	**B**	**A**	**B**	**A**	**B**
400	200	200	0	0	0	0	200	0	0	400	400	400	0	200	200	0	0	0	0	200	400
400	200	200	0	200	200	0	200	200	400	400	400	400	0	0	200	0	400	200	0	200	200
400	200	0	0	0	0	0	200	200	0	400	400	400	200	200	0	0	0	200	200	200	200
200	200	0	0	200	200	0	200	400	0	400	400	200	0	0	0	0	200	200	200	200	200
200	200	0	0	0	0	0	200	200	200	400	400	400	0	200	0	200	0	200	200	0	200
400	200	200	0	200	200	200	200	0	400	400	400	400	0	200	200	200	400	200	0	200	200
200	200	200	0	0	0	200	200	400	400	400	400	300	0	200	200	200	0	200	200	0	400
200	200	0	0	400	200	200	200	400	400	400	400	400	0	0	200	300	200	200	200	200	200
400	200	200	200	0	0	200	200	400	400	400	400	400	0	0	0	200	400	0	0	0	200
200	200	0	0	200	200	200	200	400	0	400	400	400	0	0	0	200	200	0	200	200	400
**3,000**	**2,000**	**800**	**200**	**1,200**	**1,000**	**1,000**	**2,000**	**2,600**	**2,200**	**4,000**	**4,000**	**3,700**	**200**	**1,000**	**1,000**	**1,300**	**1,800**	**1,400**	**1,200**	**1,400**	**2,600**
**Total**	**5,000**	**Total**	**1,000**	**Total**	**2,200**	**Total**	**3,000**	**Total**	**4,800**	**Total**	**8,000**	**Total**	**3,900**	**Total**	**2,000**	**Total**	**3,100**	**Total**	**2,600**	**Total**	**4,000**

The bold row indicates the sum of contribution of each individual in ten rounds. The last bold row indicates the sum of total contribution of each group which is sum of person A and B contribution.

**Table 2:** Results of 10 rounds in experiment II.

**Group 1**		**Group 2**		**Group 3**		**Group 4**		**Group 5**		**Group 6**		**Group 7**		**Group 8**		**Group 9**		**Group 10**		**Group 11**	
**(Pak Rupee)**		**(Pak Rupee)**		**(Pak Rupee)**		**(Pak Rupee)**		**(Pak Rupee)**		**(Pak Rupee)**		**(Pak Rupee)**		**(Pak Rupee)**		**(Pak Rupee)**		**(Pak Rupee)**		**(Pak Rupee)**	
**A**	**B**	**A**	**B**	**A**	**B**	**A**	**B**	**A**	**B**	**A**	**B**	**A**	**B**	**A**	**B**	**A**	**B**	**A**	**B**	**A**	**B**
400	300	100	100	100	100	100	100	1,000	100	100	1,000	100	100	100	100	100	100	200	0	0	100
200	300	100	200	100	100	100	200	0	100	200	1,000	100	0	100	200	0	100	200	0	0	100
100	300	0	0	0	0	300	100	1,000	0	100	0	200	200	200	100	100	100	0	100	100	100
400	100	200	100	100	200	100	0	0	200	200	1,000	100	300	0	0	0	200	800	0	200	100
400	300	100	100	200	200	0	100	0	0	100	0	0	100	100	100	200	200	0	600	200	0
300	400	100	200	100	100	0	300	0	100	0	1,000	100	200	200	100	200	200	0	200	100	200
0	300	0	100	100	100	100	100	100	0	0	0	200	200	100	0	100	0	200	200	100	200
400	300	200	0	200	100	100	0	100	100	100	0	100	300	0	200	200	100	400	300	200	0
400	400	100	100	100	200	0	100	0	100	100	0	0	0	100	200	100	0	0	400	100	0
400	300	100	100	0	100	200	0	100	100	100	1,000	100	100	100	100	100	200	200	200	0	200
**3,000**	**3,000**	**1,000**	**1,000**	**1,000**	**1,200**	**1,000**	**1,000**	**5,000**	**800**	**1,000**	**5,000**	**1,000**	**1,500**	**1,000**	**1,100**	**1,100**	**1,200**	**2,000**	**2,000**	**1,000**	**1,000**
**Total**	**6,000**	**Total**	**2,000**	**Total**	**2,200**	**Total**	**2,000**	**Total**	**5,800**	**Total**	**6,000**	**Total**	**2,500**	**Total**	**2,100**	**Total**	**2,300**	**Total**	**4,000**	**Total**	**2,000**

The bold row indicates the sum of contribution of each individual in ten rounds. The last bold row indicates the sum of total contribution of each group which is sum of person A and B contribution.

This experiment shows that human behavior prefers rapid rewards, as the previous section demonstrated. The results indicate that, in this case, eight groups have given us the right to use that amount for 1 week, and two groups have given us the right to use the amount for seven weeks. Only one group has given us the right to use that amount for tree plantations (see Table 3). T1 and T2 reflect intragenerational discounting, while the difference between T3 and either T1 or T2 is interpreted as intergenerational discounting. Hence, previous studies agree that the cooperation of the groups was greater when the benefits were intragenerational. Only one group opted for the tree plantation, while the other groups exhibited a selfish approach and were concerned about their own benefits in the short term. Therefore, we can say that immediate monetary rewards seem to matter the most as compared to the long-term benefits enjoyed by future generations.

**Table 3:** Intergenerational and intragenerational contributions of experiment I.

**Time period**	**T1 (one week)**	**T2 (seven weeks)**	**T3 (tree plantation)**
No. of Groups	8	2	1

The second experiment also had 22 members in 11 groups, with each group having two participants. Three out of 11 groups chose T1, three out of eleven groups chose T2, and five went with tree plantation, T3 (see Table 4). The “warm glow” or “pleasure of giving” is higher with earned income (Luccasen and Grossman, 2017), so individuals can contribute more when money is earned rather than received.

**Table 4:** Intergenerational and intragenerational contributions of experiment II.

**Time period**	**T1 (one week)**	**T2 (seven weeks)**	**T3 (tree plantation)**
No. of Groups	3	3	5

The results reflect that the motivation element absent in previous experiments can reinforce a positive approach toward climate change. When the participants were given motivation and free will, they were more inclined toward intergenerational benefits, thus choosing T3. As can be seen in experiment 1, awareness was not provided, and therefore only one group invested in the noble cause, but in experiment 2, five groups showed interest in the tree plantation. It added a new thing to previous literature: when we show people that their acts of giving can positively influence many lives, they become more concerned about climate change. So, we found that people are more inclined to contribute to future generations' benefit when convinced that their donation will make a huge and lasting positive change toward better climatic conditions. Also, when we promoted the “natural willingness to help,” we found that it led to greater spending, showing that most people believe in the value of giving and making contributions to the future of the community when they have the free will to make donations. As a result, participants were more concerned about the intergenerational benefit that tree plantations provide because it benefits future generations.

## 4. Conclusion

Climate change is an ever-present danger that is staring us in the face. Its impact is visible in various areas worldwide, including the recent flash floods in Pakistan. Developing countries are experiencing rapid environmental depletion in an attempt to boost their economic performance (Zhang et al., 2020). The problem is exacerbated by their inaction on climate change and their tendency to shift responsibility to future generations. We carried out a group experiment centered on climate change. Participants could opt to work together for a noble cause, such as tree planting for climate improvement. Its benefits are delayed for several years, but future generations will benefit greatly. There were two further options: the first was a one-week delay in reward, and the second was a seven-week delay. In experiment 1, participants were asked to contribute cash that had been granted to them, while no funding was provided in experiment 2. They were, in reality, free to give as they pleased.

Furthermore, we motivated people to help prevent dangerous climate change. We found that intergenerational discounting was high when the groups had free will and motivation. To forestall further deterioration, there is a need for concrete actions by all stakeholder groups. This study has established that the lack of knowledge and information are the main reasons for this inaction. Humans must be motivated, educated, and informed to take action to combat climate change, including tree planting. On the other hand, the provision of monetary incentives alone is not sufficient and does not automatically initiate action by human beings. Therefore, policymakers in the future should strive to increase awareness rather than incentivize climate change actions through monetary benefits.

As the extent of economic activity has continuously increased, so has the possibility of environmental concerns caused by that activity, which has crossed both geographic and generational lines. When environmental issues were smaller in scale, the nation-state was an adequate form of political organization for tackling them. While each generation used to be free to satisfy its own wants without caring about the requirements of future generations, intergenerational impacts are currently increasingly prevalent. Solving these challenges needs international cooperation. Because future generations are unable to speak for themselves, the current generation must do so. Thus, this study recommends that current policies reflect their responsibility to future generations.

## Data availability statement

The raw data supporting the conclusions of this article will be made available by the authors, without undue reservation.

## Ethics statement

The studies involving human participants were reviewed and approved by School of Social Sciences and Humanities. The patients/participants provided their written informed consent to participate in this study.

## Author contributions

SA contributed to conception and design of the study. NS and FB organized the database. SA, VS, NS, and FB performed the statistical analysis. VS, NS, and FB wrote the first draft of the manuscript. All authors contributed to manuscript revision, read, and approved the submitted version.
